# Influence of COVID-19 Lockdowns on the Usage of a Vision Assistance App Among Global Users With Visual Impairment: Big Data Analytics Study

**DOI:** 10.2196/26283

**Published:** 2021-05-12

**Authors:** Gang Luo, Shrinivas Pundlik

**Affiliations:** 1 Schepens Eye Research Institute Massachusetts Eye and Ear Harvard Medical School Boston, MA United States

**Keywords:** assistance, assistive technology, COVID-19, development, eye, low vision, needs, smartphone apps, usage, vision assistance, vision, visual impairment

## Abstract

**Background:**

Millions of individuals with visual impairment use vision assistance apps to help with their daily activities. The most widely used vision assistance apps are magnifier apps. It is still largely unknown what the apps are used for. Lack of insight into the visual needs of individuals with visual impairment is a hurdle for the development of more effective assistive technologies.

**Objective:**

This study aimed to investigate how needs for visual aids may vary with social activities, by observing the changes in the usage of a smartphone magnifier app when many users take breaks from work.

**Methods:**

The number of launches of the SuperVision Magnifier app was determined retrospectively from 2018 to 2020 from among active users worldwide. The fluctuation in app usage was examined by comparing weekday vs weekend periods, Christmas and new year vs nonholiday seasons, and COVID-19 lockdowns vs the easing of restriction during the pandemic.

**Results:**

On average, the app was used 262,466 times by 38,237 users each month in 2020 worldwide. There were two major trough points on the timeline of weekly app usage, one aligned with the COVID-19 lockdowns in April 2020 and another aligned with the Christmas and new year week in 2018 and 2019. The app launches declined by 6947 (11% decline; *P*<.001) during the lockdown and by 5212 (9% decline; *P*=.001) during the holiday weeks. There was no significant decline during March to May 2019. App usage compensated for seasonal changes was 8.6% less during weekends than during weekdays (*P*<.001).

**Conclusions:**

The need for vision assistance technology was slightly lower during breaks and lockdowns, probably because the activities at home were different and less visually demanding. Nevertheless, for the entire user population, the needs for visual aids are still substantial.

## Introduction

People with impaired visual acuity typically use magnifying devices for a wide range of daily reading tasks. As smartphones and tablets have become popular in recent years, there has been an increase in the use of vision assistance mobile apps, which utilize embedded cameras and turn mobile devices into handheld electronic magnifiers. As the mobile devices are already at hand, their convenience, together with the benefits of electronic magnification, such as an adjustable zoom level, have provided significant advantages over traditional optical magnifiers. The mobile apps are increasingly used by young [1] and older individuals with visual impairment [2]. A recent survey reported that >50% of people with visual impairment use smartphone magnification apps [3]. Our recent study with more than 16,000 magnifier app users reported that they mostly use the app for short spot-reading tasks [4]. This finding is consistent with a view accepted among those with low vision: magnifier use is not limited to reading only long-passage text. Some studies have focused on spot reading in activities of daily living, such as finding phone numbers and reading instructions on medicine bottles [5-8].

However, what constitutes the spectrum of activities for which people are using mobile magnifier apps is still an open question. Understanding the purposes of app use can be significant for low vision research, because technology development and evaluation ideally should respond to the actual, real-life needs of users. Otherwise, a visual aid, although reportedly useful, might not be as useful in everyday life.

The important knowledge about the needs of individuals with visual impairment for vision assistance include, albeit not exclusively, the purpose of visual tasks (eg, mobility and leisure reading), visual targets (eg, text, object, and pet), environment (eg, location and lighting conditions), effectiveness (or gap), and the availability of vision assistance means (eg, magnifiers and sighted companions). It should be noted that their needs may evolve dynamically. Starke et al [9] recently classified the visual needs of 32 people with low vision on the basis of images captured by body-mounted cameras over a 1-week period. They reported that individuals with visual impairment frequently need help with using mobile devices, which were not widely available 10 years ago.

This study reports an important finding that can contribute to our understanding of the visual needs of smartphone magnifier app users. Specifically, this study aimed to investigate whether vision assistance app users are those who are mostly confined at home owing to vision impairment. Our goal is to find evidence indicating that some of app usages are involved in social activities outside of home.

Instead of a direct approach to interview users, this study harnessed a global event, the COVID 19 pandemic, as an unexpected intervention to deduce the underlying purpose of magnifier app uses. The approaches to directly observe users’ behaviors can result in less ambiguous data, but it is usually very challenging to enroll a large sample of participants owing to the potential introduction of a sampling bias. Our chosen method studies the entire app user population as a black box. While the study has a limitation with regard to the lack of an experimental control, obtaining big data from tens of thousands of users has the potential to reveal larger population-level trends and may yield valuable findings.

Specifically, if a significantly large number of magnifier app users are not confined at home under normal circumstances and go to work or school, app usage is expected to decrease during the COVID-19 lockdown. On the contrary, if the magnifier apps are mostly used for household activities at home, app usage is not expected to decrease but rather increase during the lockdown, as people spend more time at home. As this study is related to social activity, we also investigated app usage during weekends and holidays, with the same expectation of observing the same effect as that during the lockdown.

## Methods

### SuperVision Magnifier App and Users

Data presented here were collected for 38 weeks, from November 2019 to July 2020, using the freely available SuperVision Magnifier app, which was developed by our group. The iOS version of the SuperVision Magnifier (version 1.7.16 used in this study) was released worldwide in October 2016. The app provides vision assistance features, which are commonly available in other smartphone magnification apps and dedicated handheld video magnifiers: up to 16× digital zoom, the ability to capture a snapshot, color inversion (and binarizing images), and a toggling flashlight. The app can be considered a representative of many other similar magnifier apps available in the App store.

Data were collected from active users worldwide, which included 92% of returning users, who repeatedly used the app at different frequencies, and 8% of new users, who installed the app for the first time. Because the log data of 1 app launch are always uploaded to the server when the app is opened the next time, those new users must have used the app at least twice. A new user becomes a returning user if he/she launches the app at least 1 day after the first time. People who installed the app but did not use it during the period of data collection were not included in this study.

### Data Collection Methods

A data collection module is embedded in the app to collect runtime usage data. Each time the app became the foreground app, a launch event was recorded. The data logging module was developed using the analytics software development kit of Umeng (Beijing, China). Data were received in a consolidated form from the whole user group, and the users’ privacy was not compromised as no participant-identifiable information was collected. The study was approved by the institutional review board of the Massachusetts Eye and Ear Infirmary and was exempt from the regulations of studies on human subjects because the aggregated data prevented the identification of participants.

To further obtain direct evidence regarding the geolocations where the app is used, we released a new version (1.8.1) in September 2020, in which the distance between 2 consecutive app launches is calculated and uploaded to the Umeng server. The specific geolocation data are not saved because of the requirement of privacy protection. These types of data would indicate whether the app is used only at the same location.

For user activity analysis, consolidated weekly app usage reports were downloaded from the website of the analytics service provider, Umeng. For the timeline of major events related to the COVID-19 pandemic, information was extracted from several reliable websites [10-15].

### Statistical Analysis

Statistical analysis was performed using the time series data for global weekly app launches from November 2019 to July 2020. Since the time series observations are not independent, autoregressive integrated moving average (ARIMA) modeling was used for analysis. The effect of COVID-19 lockdowns on magnifier app usage was analyzed using an ARIMA model, which included a predictor that indicated the presence of an intervention; that is, the occurrence of COVID-19 lockdowns. A similar but separate analysis was conducted to determine the effect of holidays on app usage.

The following segments of the weekly app launch data time series were identified: (1) “Holidays”: a 2-week duration related to Christmas and new year’s day; (2) “Prelockdown”: the weeks before March 15, 2020, excluding holidays, which were assumed to represent normal app usage in the absence of interventions; (3) “Lockdown”: 8 weeks starting from the week of March 15 to May 10, 2020; and (4) “Postlockdown”: the weeks after May 10, 2020. Given that the pandemic affected different countries and regions worldwide at slightly different times, the rationale for choosing the period between March 15 and May 10, 2020, as the lockdown period was that these 8 weeks coincided with the spread of the pandemic across a large part of the world, including North America, Europe, and Asia.

The effects of lockdown and holiday interventions on app usage were analyzed separately. When analyzing the effect of holidays, only the prelockdown and holidays segments of the time series were considered. When analyzing the effect of lockdowns, only the prelockdown and lockdown segments were considered.

Statistical analysis was performed using the ARIMA function from the standard library in R (The R Foundation), along with associated functions for evaluating and diagnosing the fitted model. Coefficient (*β*) values, SE, and *P* values are reported when describing the effect of the interventions. An effect with a *P* value of <.05 was considered significant.

Because the active users of the app grew over time, when investigating app usage during weekends, the number of daily app launches was normalized by the weekly average to exclude the effect of the growing trend. This measure could also compensate for the seasonal changes because of holidays and COVID-19 lockdowns in this study.

## Results

**Figure 1:**
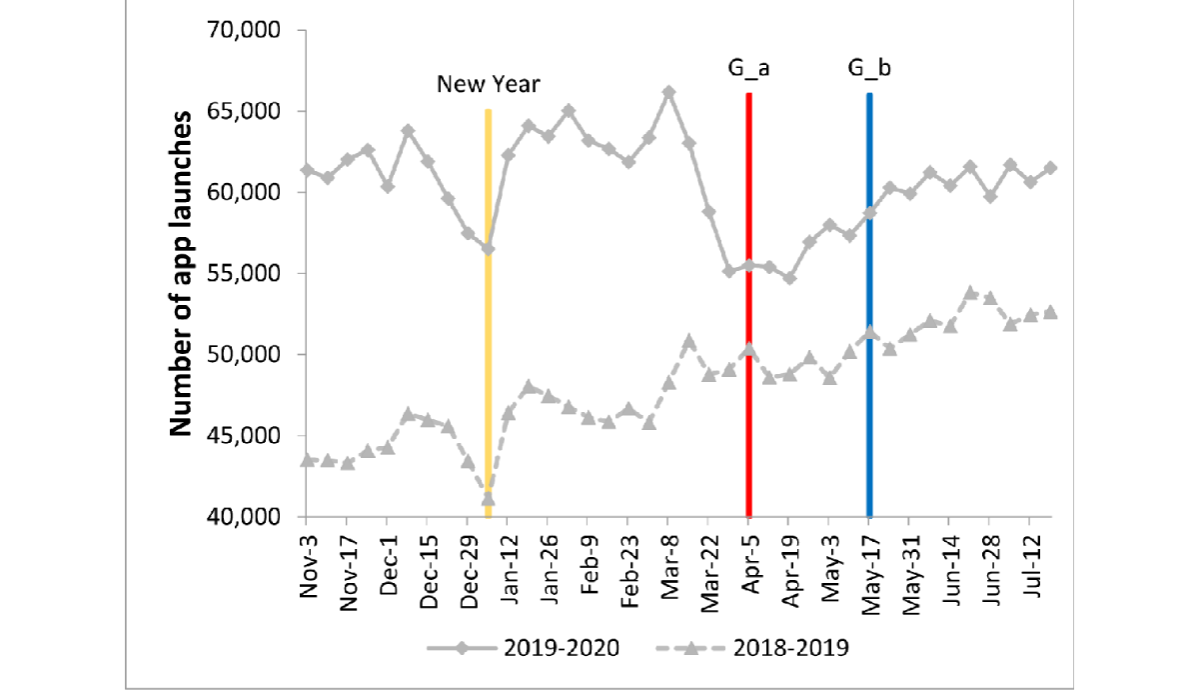
Weekly app launches (number of times of app usage) from November 2019 to July 2020, compared to the previous year’s data for the same duration (dotted line). For the curve representing November 2019 to July 2020, there are 2 troughs around new year’s day and COVID-19 lockdowns in April 2020. A corresponding trough near new year’s day 2019 is observed in the previous year’s curve, but there is no corresponding decline in April 2019. G_a: approximately one-third of the world's population is living with COVID-19 restrictions, G_b: some countries, including Spain, Iran, Italy, Denmark, Israel, Germany, New Zealand, and Thailand began to ease lockdown restrictions.

Between November 2019 and July 2020, the app was used 262,466 (SD 10,277) times by 38,237 (SD 983) users each month and 60,505 (SD 2,877) times by 14,585 (SD 474) users each week on average, worldwide. On average, each user launched the app 4.1 (SD 0.1) times per week. The solid curve in Figure 1 shows the fluctuation in weekly app launches from November 2019 to July 2020. There are 2 major troughs in the curve, which appear to be aligned with the new year holiday and COVID-19 lockdowns. The number of app uses decreased from the preholiday weekly peak of 63,807 to 56,510 during the holiday weeks, and it decreased from the prelockdown peak of 66,182 to the lowest point at 54,702 during the lockdowns. As some countries started to ease lockdown restrictions, the weekly app launches rose to 58,730 in mid-May 2020, and the recovery appears to have continued through July 2020. To exclude the possibility that the decline during the lockdown period was due to a seasonal change and not lockdowns, the weekly app launches from November 2018 to July 2019 are also plotted as a dotted curve in Figure 1. As shown in Figure 1, there was a decline in app launches around the new year holiday of 2019, but there was no major decline in April 2019. This observation suggests that the decline during April 2020 was not seasonal but rather possibly due to the COVID-19 lockdowns.

An ARIMA (0,1,1) model with interventions was fitted to the weekly app launch data. The effect of lockdowns on app use was found to be significant (*β*=–6947, SE=1039; *P*<.001). The negative value of the coefficient for the lockdown variable indicates that the number of app launches decreased by an average of 6947 per week (approximately 11%) during the lockdown compared to the prelockdown phase. Similarly, the intervention resulting from holidays had a significant effect (*β*=–5212, SE=1307; *P*=.001). Again, the negative value of the coefficient indicates that the weekly average app launches decreased by 5212 during the holiday weeks.

From November 2018 to July 2019, there was a significant reduction in app launches around the holiday weeks of Christmas and new year’s day (*β*=–2168, SE=720; *P*=.003). However, there was no significant change in the number of app launches per week during mid-March to early May 2019 (*β*=–643, SE=603; *P*=.29).

**Figure 2:**
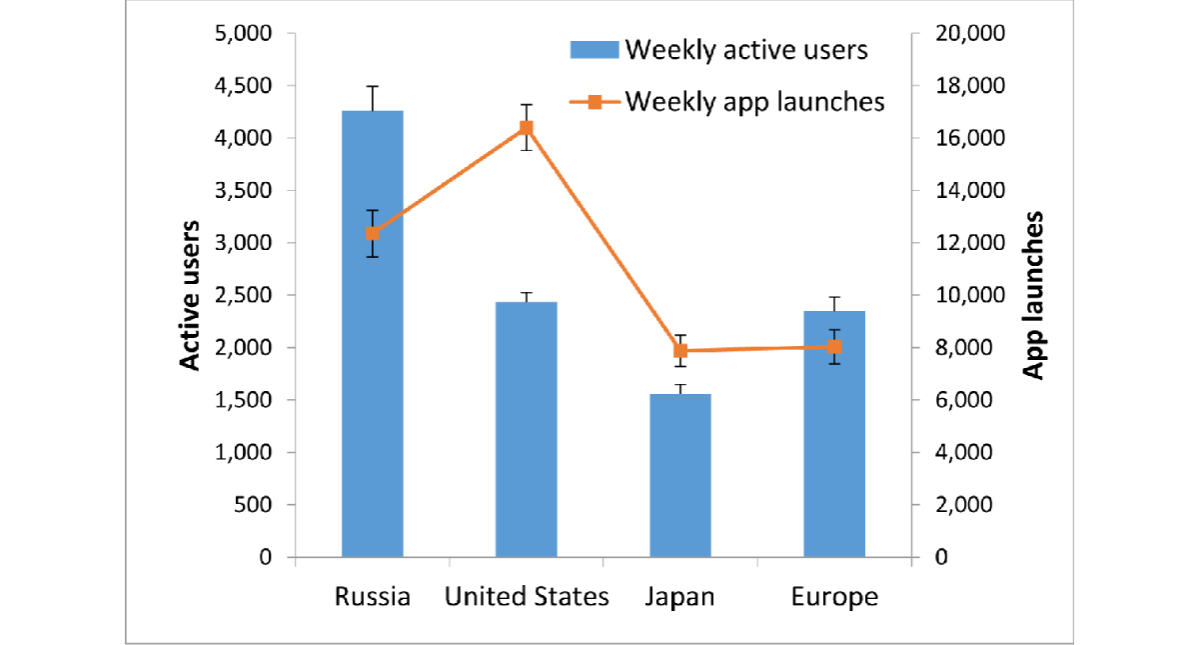
Four regions were included for separate analysis: Russia, the United States, Japan, and Europe. Their average numbers of weekly active users were 4259, 2436, 1558, and 2346, respectively. The corresponding average numbers of weekly app launches (number of times the app was used) were 12,353, 16,393, 7880 and 8031, respectively. Error bars indicate SD values.

Since the pandemic’s course was not the same across all countries, the fluctuation in app usage was also visualized by country or region to confirm that the patterns observed in the global data could still be observed in each country or region. App users were located in more than 120 countries, but most of the countries had only a small number of users. We selected four countries or regions with most users for further analysis: Russia, the United States, Japan, and Europe (Figure 2). There were more than 1500 active users weekly in each of these selected regions. No other country had an average of more than 588 weekly users.

**Figure 3:**
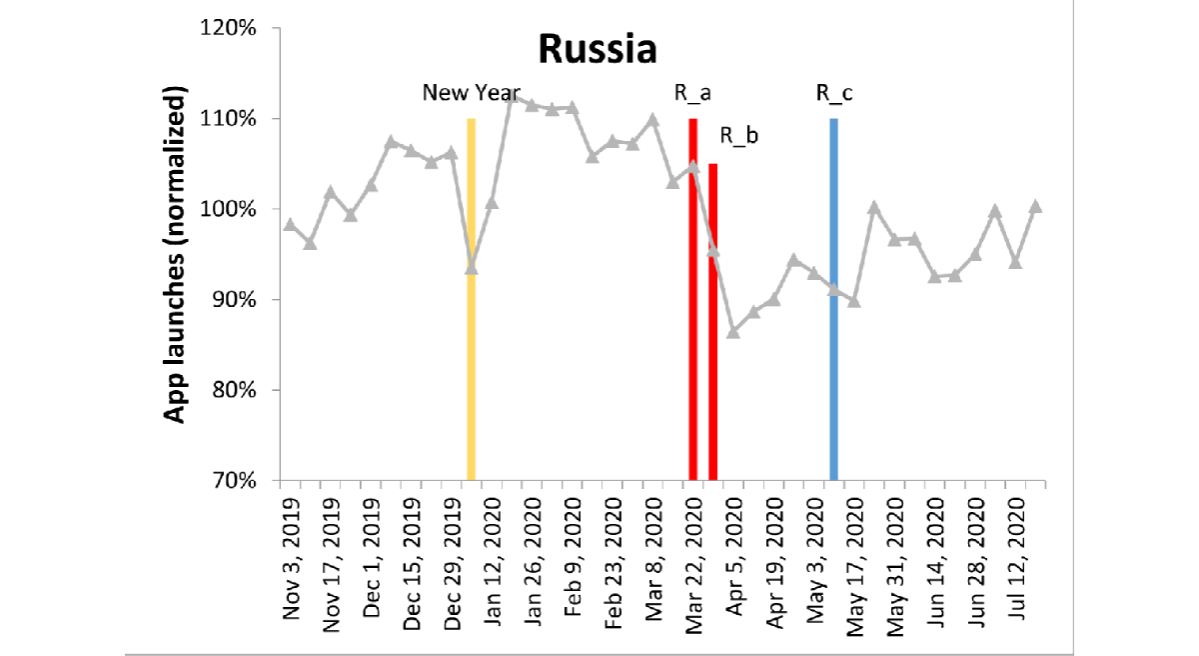
App launch fluctuations in Russia, normalized by the average number of weekly app uses. Yellow: holiday, red: lockdown, and blue: postlockdown. In Russia, Eastern Orthodox Christmas was on January 7. R_a: Ministry of Culture announced the closure of all cultural institutions under its jurisdiction; Minister of Education Sergey Kravtsov announced the closure of all Russian schools from March 23, 2020. R_b: Moscow issued a stay-at-home order for all residents. R_c: President Vladimir Putin announced an end to Russia’s 6-week nationwide lockdown despite a record increase in COVID-19 cases.

**Figure 4:**
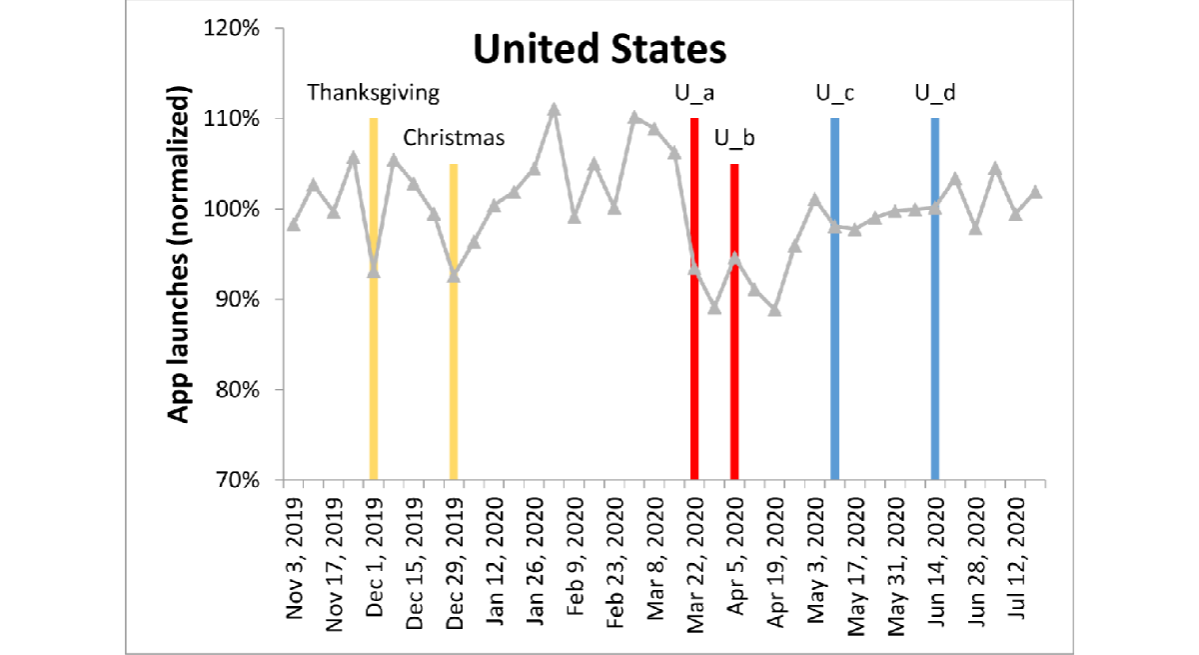
App launches in the United States, color-coded as in Figure 3. U_a: New York City public schools closed, and California issued a stay-at-home order for all 40 million residents. U_b: approximately 91% of people in the United States were instructed to stay at home. U_c: Florida and California reopened. U_d: New York City began phase I of reopening.

**Figure 5:**
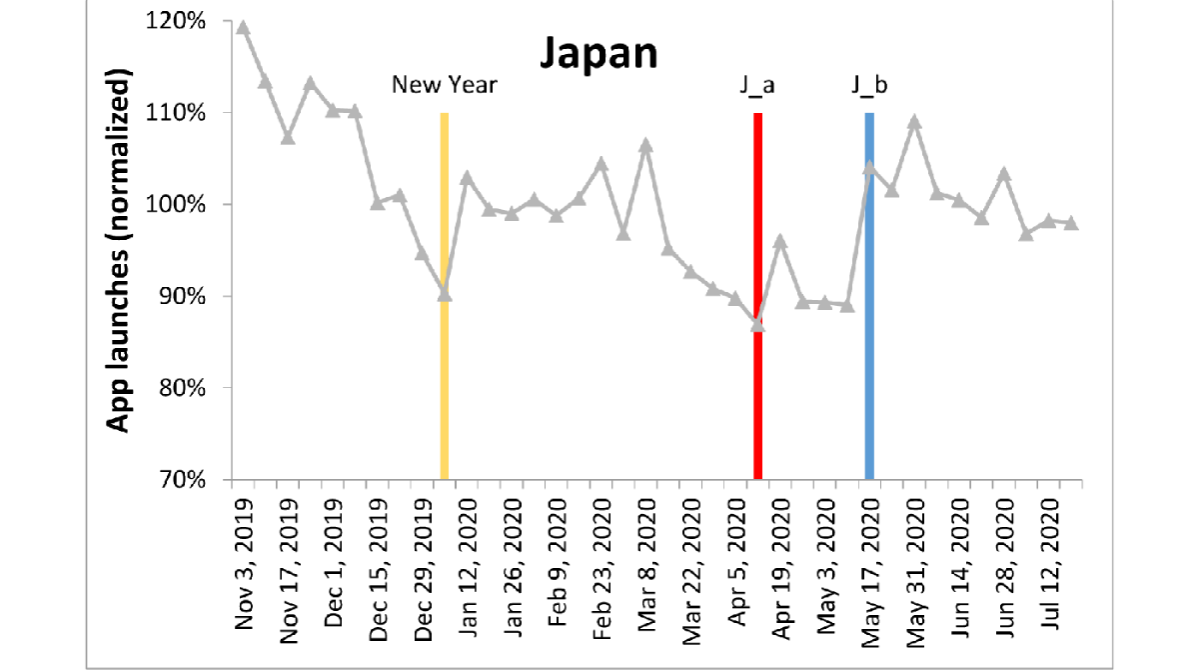
App launches in Japan, color-coded as in Figure 3. J_a: Japan’s prime minister proclaimed a 1-month state of emergency from April 8 to May 6, 2020, for Tokyo and the prefectures of Kanagawa, Saitama, Chiba, Osaka, Hyogo, and Fukuoka. J_b: Japan’s prime minister lifted the state of emergency imposed in 39 of 47 prefectures, announcing that the nation’s rate of infection has decreased to one-seventh of its peak.

**Figure 6:**
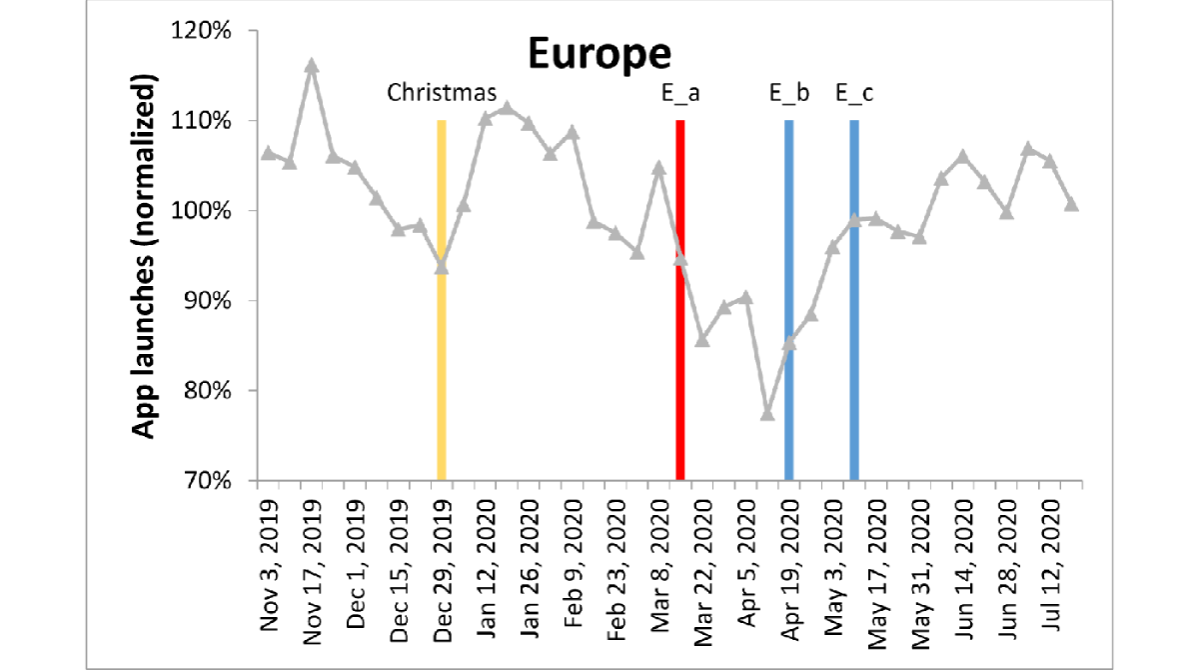
App launches in Europe, color-coded as in Figure 3. E_a: All shops and venues closed nationwide in Italy. E_b: Economy reopened in Germany. E_c: Lockdown restrictions started to be eased in several countries such as Spain, Italy, Denmark, and Germany.

Figures 3-6 show the fluctuation in weekly app launches during a period of 38 weeks from November 2019 to July 2020 in the aforementioned 4 regions. The timeline of some major events are superimposed on the fluctuation curves. Three types of events are color-coded: yellow=holiday, red=lockdown, and blue=easing of lockdown restrictions. It can be seen that Christmas or new year are aligned with one of the major troughs in the 4 regions. For the United States, there seemed to be an additional holiday trough around Thanksgiving.

Consistently, the lockdown events in these 4 regions were either during the major trough in the global data or during the decreasing phase shortly before that trough. Similarly, the regional lockdown easing events occurred during the increasing phase of global app use.

**Figure 7:**
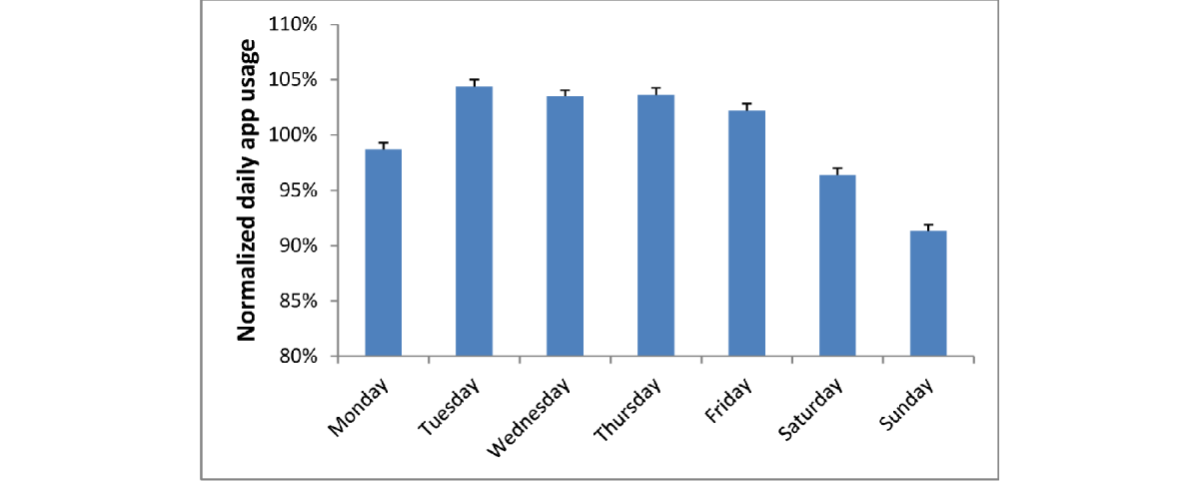
Daily app launches normalized by the weekly average for the period between November 2019 and July 2020. Bars indicate mean app usage by the days of the week. Error bars indicate SD values.

Figure 7 shows the normalized daily app usage by days of the week between November 2019 and July 2020. Overall, app usage on weekends was 8.6% lesser than that on weekdays (93.9% vs 102.5%, respectively; *P*<.001). As shown in Figure 7, app usage was the lowest on Sunday. App usage on Saturday and Monday, which are similar to that on Sunday, were also lower than the that during other weekdays. Because app users were at different time zones worldwide, the local time for most users differed from the time of the Umeng analytic server (GMT+8 hours). Therefore, the effect of the weekend spills over 1 day before and 1 day after. If Saturday and Monday are excluded, usage on Sunday was 12% lesser than the average usage from Tuesday to Friday.

**Figure 8:**
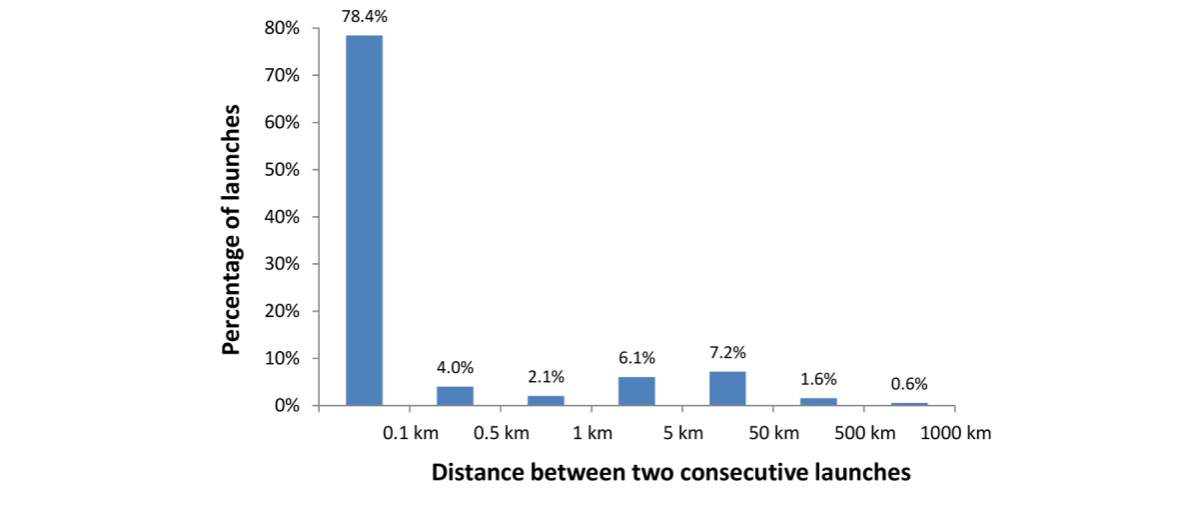
Percentage of app launches vs the distance to the location of the previous launch. Most consecutive app launches occurred at the same place. Excluding the bin for the same location, there is a sizeable peak between 5 km and 50 km, which indicates that 7.2% of the consecutive app launches were separated by a distance of 5-50 km.

Figure 8 shows the distribution of the distance between 2 consecutive app launches. The data were collected across a period from September 15, 2020, to February 16, 2021, including total of 414,618 samples. Far from being restricted to 1 place, a small number of users travelled more than 500 km. Most consecutive app uses were recorded at the same locations, but this does not necessarily indicate that 1 pair of (consecutive) app launches occurred at the same location as that of another pair of (consecutive) app launches. A sizeable peak was observed between 5 km and 50 km (7.2%). For reference, the average commute distance in the United States is 24 km each way [16].

## Discussion

### Principal Findings

This study investigated the impact of changes in social activities on the app launch pattern of tens of thousands of SuperVision Magnifier app users and observed a declining trend in app usage corresponding to weekends, major holidays, and the initiation of lockdowns in different regions compared to the prelockdown usage frequency. Given the black box of the mixed user population, the global COVID-19 lockdown effectively provided a window through which insights can be gained by investigating the effect of a behavioral intervention, which is not planned or designed. In this study, this opportunity was utilized to help deduce the situations where the vision assistance app was used.

Our previous study reported that the mobile magnifier app is mostly used for short spot-reading, while a small number of users may use the app for long passage–reading for ≥30 minutes each time [4]. In terms of app launch, a small portion of users used the app many times on a daily and weekly basis, and many users used it only occasionally. It should be noted that the number and duration of app uses are not necessarily a measure of helpfulness. Those metrics are typically used to gauge the customer adherence to video game and social media apps. For vision assistance apps, those metrics reflect the needs of users (especially returning users). For instance, Google Maps is very useful for traveling to unfamiliar places, but many people do not use it every day. In addition to launch and duration measures, our latest study on visual targets viewed by users through the app reported that more than half of the targets are nontextual targets including indoor and outdoor contexts, food, animals, and plants [17]. Furthermore, our study shows that the locations, where vision assistance is needed, are not restricted to home. Together, the wide range of frequency, the variety of target categories, and the effects of social activity–related locations suggest that the magnifier app can probably meet many different visual needs in the daily lives of people with visual impairment. Our ongoing studies are attempting to generate an overview of the visual needs of these individuals from different perspectives.

It remains unknown what factors influence the needs or in what context those visual tasks are performed with help from the app. Without fully understanding the actual visual needs in the real world, limited studies on low vision and technologic developments have been relying on subjective opinions and limited surveys. Thus far, previous studies have evaluated the spot reading performance of vision assistance interventions based on visual tasks that are typically carried out at home [5,7], including reading information on medicine bottles, product labels, and utility bills, all of which are textual targets. However, visual tasks outside of home (eg, at work, school, or during traveling) have been rarely considered. If we assume that the magnifier app is mostly used at home, app use would have increased during lockdowns; however, we observed the opposite trend from the app usage data.

The data on distance between 2 consecutive app launches directly indicate that the magnifier app is also frequently used outside home. Interestingly, the travel distance seemed to be in line with the average commute distance in the United States. While our study population was large, detailed information on geolocations could not be collected. However, considering a consistent reduction in app usage globally during weekends, holidays, and COVID-19 lockdowns, it is a reasonable speculation that the reduction could be due to reduced work- or school-related activities. If this association is accurate, it would suggest that normally the magnifier app is used not only at home for activities of daily living but also at workplaces, schools, or while traveling. Regarding the proportion of app usage away from home, this study could not collect direct data. The 11% decline during lockdowns and 8.6% of decline on weekends might be a rough indicator of the frequency of such usage. According to anecdotal reports we received, the app was used by some people to read road signs or to watch presentations in conferences. However, these are only a few examples of app use outside of home. More precise estimation will be conducted in future studies.

A counter argument might be made that the reduction in app usage during COVID-19 lockdowns may not necessarily be due to a reduction in work- or school-related activities but instead, it could be simply because the users had less chances to use the app when staying at home during lockdowns. This study did not identify the environment in which the app was used, but the hypothesis cannot explain the reductions during holidays and weekends. The time people spend outside their home for traveling, socializing, outing, and shopping during these breaks should be more than that during COVID-19 lockdowns. More activities outside home during weekends in 2019 and Christmas and new year holiday weeks in 2018 and 2019 did not appear to increase app usage. On the other hand, if the decline during holidays and weekends were to be explained by less time spent at home, assuming that the magnifier app is primarily used for activities of daily living at home, then the explanation based on the time staying at home would not be able to account for the decline during lockdowns. Taken together, a more plausible explanation could be that decreased app usage during weekends, holidays, and lockdowns are due to reduced work- or school-related activities carried out away from home. As many people worked from home and many students practice remote learning during the lockdowns, it is possible some users still use the vision assistance app at home to help with their work. This study could not distinguish this portion from that of nonwork- or nonschool-related app usages. If this were true, it would imply that the app is normally used even outside home when there is no lockdown.

Another possible reason for the reduction in app usage may be that when users with visual impairment were at home, their family members and friends could help with some of the activities of daily living, or the visual tasks at home was less demanding overall than those at workplaces; hence, they did not need to use the app in some cases. Nevertheless, for the whole user population, the need for vision assistance devices on a daily basis is still substantial, as the reduction in app usage during breaks was only approximately 10%.

To confirm the aforementioned speculations, future studies are required to collect direct evidence indicating the detailed nature of activities for which the app was used, and to conduct some necessary surveys (eg, on demographics and vision status) to help interpret the behavioral data. Further big data analysis studies will help reveal the wide spectrum of visual demands for vision assistance among people with visual impairment. This knowledge will be important for developing personalized, more effective solutions in future low vision rehabilitation research. The development of vision assistance technologies can target a particular group of users (eg, elderly individuals) or the entire cohort. Whatever the targeted group is, ideally, the composition of visual tasks should roughly match the spectrum of visual needs in the targeted cohort’s actual visual needs. Otherwise, studies may miss important needs while over-representing others. Without accurate feedback from appropriate evaluation studies, the utility of technologies may be compromised.

### Conclusions

Considering our large user sample, this longitudinal study observed a slight reduction in the usage of the SuperVision Magnifier app during weekends, holidays, and COVID-19 lockdowns. We surmise that the possible reasons could be that the magnifier app is normally used in some work- and school-related activities in addition to daily tasks performed at home, and the visual tasks at home may be relatively less demanding than those performed outside of home.

## Abbreviations

ARIMA: autoregressive integrated moving average

## References

[1] Chun R, Bhakhri R, Coalter J, Jay WM (2012). Smartphone Usage in Patients with Optic Atrophy. Neuro-Ophthalmology.

[2] Bhakhri R, Chun R, Coalter J, Jay W (2012). A Survey of Smartphone Usage in Low Vision Patients. Invest Ophthalmol Vis Sci.

[3] Crossland MD, Silva RS, Macedo AF (2014). Smartphone, tablet computer and e-reader use by people with vision impairment. Ophthalmic Physiol Opt.

[4] Luo G (2020). How 16,000 people used a smartphone magnifier app in their daily lives. Clin Exp Optom.

[5] Wittich W, Jarry J, Morrice E, Johnson A (2018). Effectiveness of the Apple iPad as a Spot-reading Magnifier. Optom Vis Sci.

[6] Pundlik S, Singh A, Baghel G, Baliutaviciute V, Luo G (2019). A Mobile Application for Keyword Search in Real-World Scenes. IEEE J Transl Eng Health Med.

[7] Owsley C, McGwin G, Sloane ME, Stalvey BT, Wells J (2001). Timed instrumental activities of daily living tasks: relationship to visual function in older adults. Optom Vis Sci.

[8] West SK, Rubin GS, Broman AT, Muñoz B, Bandeen-Roche K, Turano K (2002). How does visual impairment affect performance on tasks of everyday life? The SEE Project. Salisbury Eye Evaluation. Arch Ophthalmol.

[9] Taylor DJ, Hobby AE, Binns AM, Crabb DP (2016). How does age-related macular degeneration affect real-world visual ability and quality of life? A systematic review. BMJ Open.

[10] Neilson S, Woodward A (2020). A comprehensive timeline of the coronavirus pandemic at 1 year, from China's first case to the present. Insider.

[11] Kantis C, Kiernan S, Bardi J (2021). UPDATED: Timeline of the Coronavirus. Think Global Health.

[12] COVID-19 pandemic in Japan. Wikipedia.

[13] Xue F (2020). Japan's Response to the Coronavirus: A Timeline of Major Events. University of Virginia: College and Graduate School of Arts and Sciences.

[14] COVID-19 pandemic in Russia. Wikipedia.

[15] Harding L (2020). Global report: Russia to end national lockdown as French shops reopen. The Guardian.

[16] (2003). From Home to Work, the Average Commute is 26.4 Minutes. OmniStats.

[17] Luo G (2021). What Visual Targets Are Viewed by Users With a Handheld Mobile Magnifier App. Trans Vis Sci Tech.

